# Macrodactyly of the Foot in a Newborn: A Case Report and Four-Year Follow-Up

**DOI:** 10.7759/cureus.114333

**Published:** 2026-08-11

**Authors:** Maria Bandeira Duarte, Marta Valerio, Ana Filipa Vilarinho

**Affiliations:** 1 Pediatrics Department, Hospital de Santarém, Unidade Local de Saúde da Lezíria (ULS Lezíria), Santarém, PRT

**Keywords:** clinical evolution, congenital deformity, macrodactyly, multidisciplinary approach, surgical treatment

## Abstract

Introduction: Macrodactyly is a rare congenital malformation characterized by the overgrowth of all mesenchymal elements in one or more digits of the hands or feet. Although diagnosis typically occurs at birth, prenatal detection via ultrasound has been reported. The condition imposes a significant psychological burden on children and their families. Pediatricians are often the first clinicians to inform and counsel anxious parents; accurate recognition of this malformation and clear communication regarding the multidisciplinary approach, clinical evolution, and overall prognosis are therefore essential.

Case presentation: A full-term female neonate presented at birth with an isolated enlargement of the second and third digits of the left foot. There were no other abnormalities. A family history of a structural hand anomaly in the maternal grandmother was noted. After 4.5 years of follow-up, the asymmetry progressively accentuated, consistent with the progressive type of macrodactyly.

Conclusions: Macrodactyly requires early recognition and long-term multidisciplinary management involving pediatricians, orthopedists, geneticists, physiatrists, and surgeons. Surgical intervention is typically inevitable, and maintaining realistic expectations is crucial. Despite the challenges, the overall prognosis is favorable, with no adverse effects on systemic growth or neurodevelopment.

## Introduction

Macrodactyly is a congenital enlargement of one or more digits of the hands or feet [1-4]. It is characterized by the overgrowth of all mesenchymal elements--a feature that defines "true macrodactyly"--and distinguishes it from conditions involving the enlargement of a single tissue type, such as hemangiomas, lymphangiomas, or enchondromas [1,4-7].

It is a rare malformation with an estimated prevalence ranging from 1 in 50,000 to 1 in 200,000 live births [2,3]. Macrodactyly may occur as an isolated finding or as a clinical manifestation of systemic syndromes, including Proteus syndrome, Bannayan-Riley-Ruvalcaba syndrome, Maffucci syndrome, Klippel-Trénaunay-Weber syndrome, neurofibromatosis type 1, Ollier disease, and Milroy disease [1-4]. These associations must be ruled out through complementary diagnostic testing and clinical surveillance [1-4]. In isolated cases of true macrodactyly, heredity does not appear to be involved [5]. Although its pathogenesis is not yet fully elucidated, somatic mutations in PIK3CA have been implicated in its etiology [2,3,8,9]. Timing of mutation during development and the affected tissue lead to distinct disease syndromes, which is reflected by the broad phenotypic spectrum--PIK3CA-related overgrowth spectrum (PROS) [10].

Both hands and feet may be affected [1,3,5]. While some authors suggest a higher frequency in the feet [1], others report a predominance in the hands [3] or find no significant difference in distribution [4]. Although any digit may be involved--either in isolation or affecting multiple adjacent digits--the second and third digits are most frequently affected [1,4,7,8].

The clinical presentation is unique to each patient, necessitating regular monitoring to evaluate the growth rate through objective measurements [3]. Two distinct clinical types are recognized: static and progressive [3-5,7]. In the static type, the enlarged digit grows proportionately with the child's overall development, maintaining a consistent disproportion [5]. In the progressive type--which is more common--the affected digit exhibits accelerated growth relative to unaffected tissues, progressively accentuating the deformity over time [5].

Diagnosis typically occurs at birth, though prenatal detection via ultrasound has been documented [1,5]. The condition imposes a significant psychological burden on children and their families, arising from aesthetic concerns, physical discomfort, chronic pain, functional impairment, and psychosocial challenges [2,3,5-8]. A common early complaint is a notable discrepancy in shoe size or the requirement for customized footwear [3,7]. Despite these challenges, the overall prognosis is favorable, with no adverse effects on systemic growth or neurodevelopment.

## Case presentation

A full-term female neonate was born to a primigravida mother following an unremarkable pregnancy and an uncomplicated vaginal delivery. The mother denied tobacco, alcohol, or recreational drug use, reporting only standard prenatal supplementation. Antenatal ultrasounds were normal, and there was no evidence of consanguinity. Notably, the family history revealed a structural hand anomaly in the maternal grandmother.

**Figure 1:**
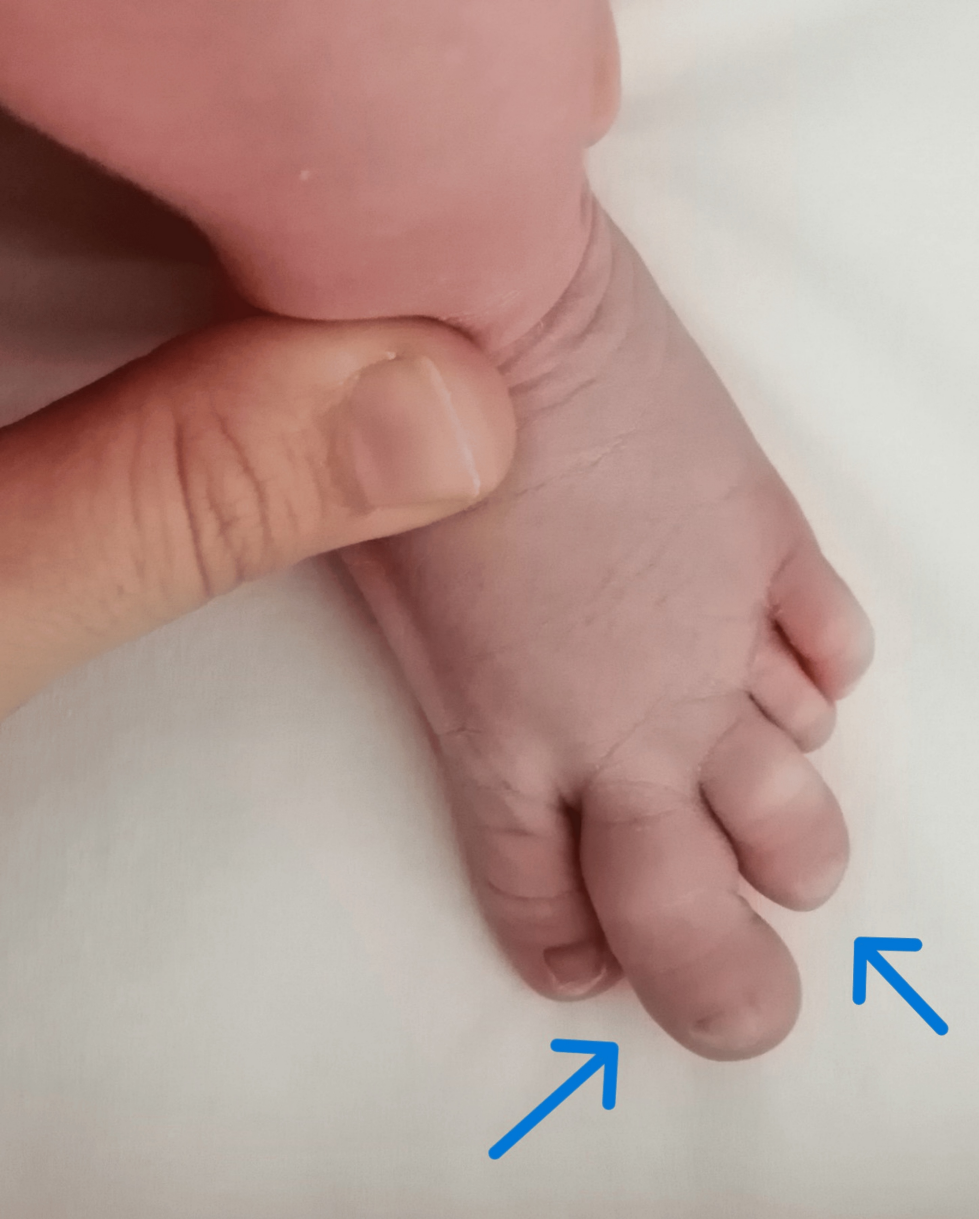
Macrodactyly involving the second and third digits of the left foot; clinical appearance of the left foot on the third day of life.

**Figure 2:**
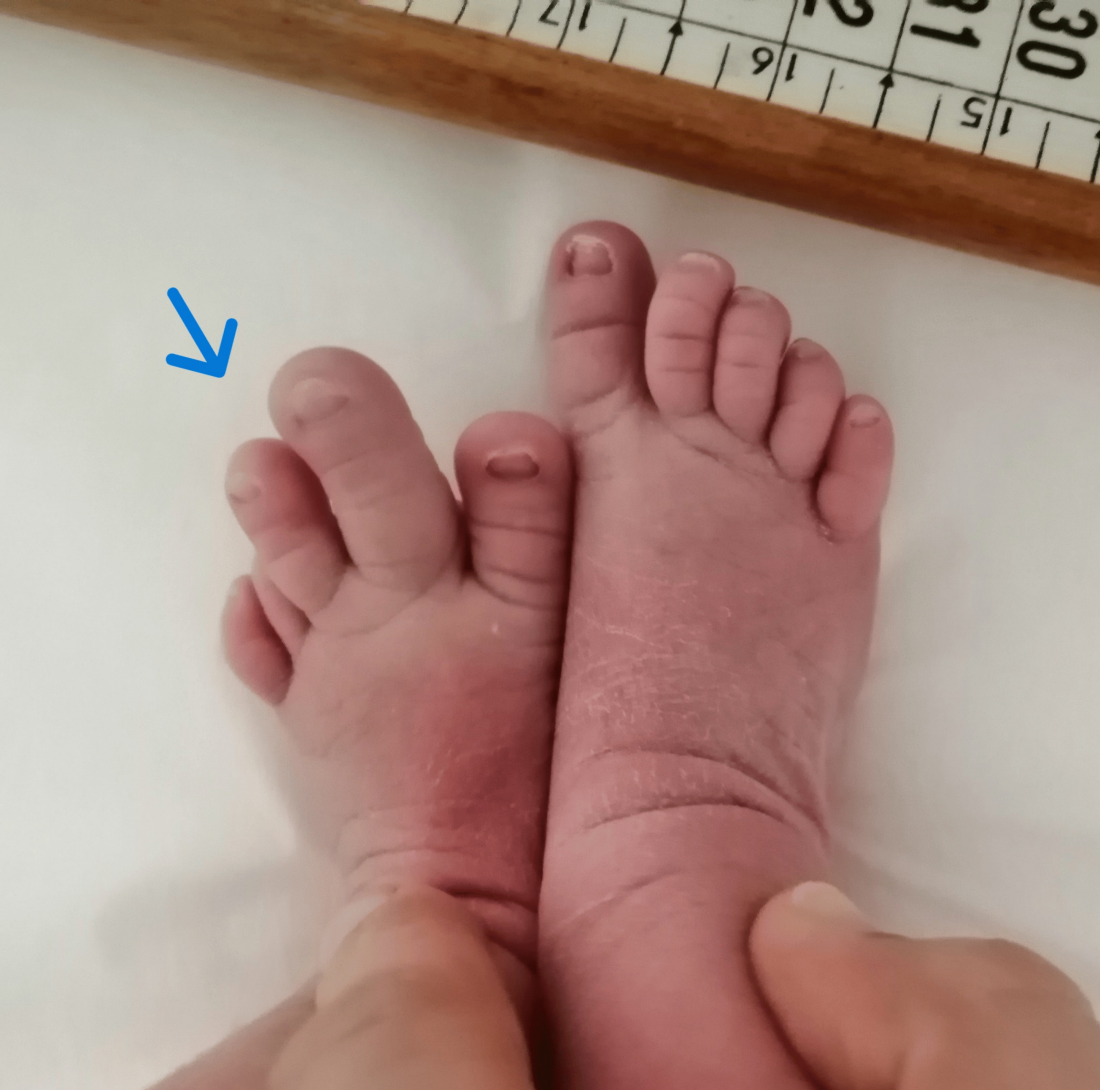
Macrodactyly involving the second and third digits of the left foot resulting in significant feet asymmetry.

**Figure 3:**
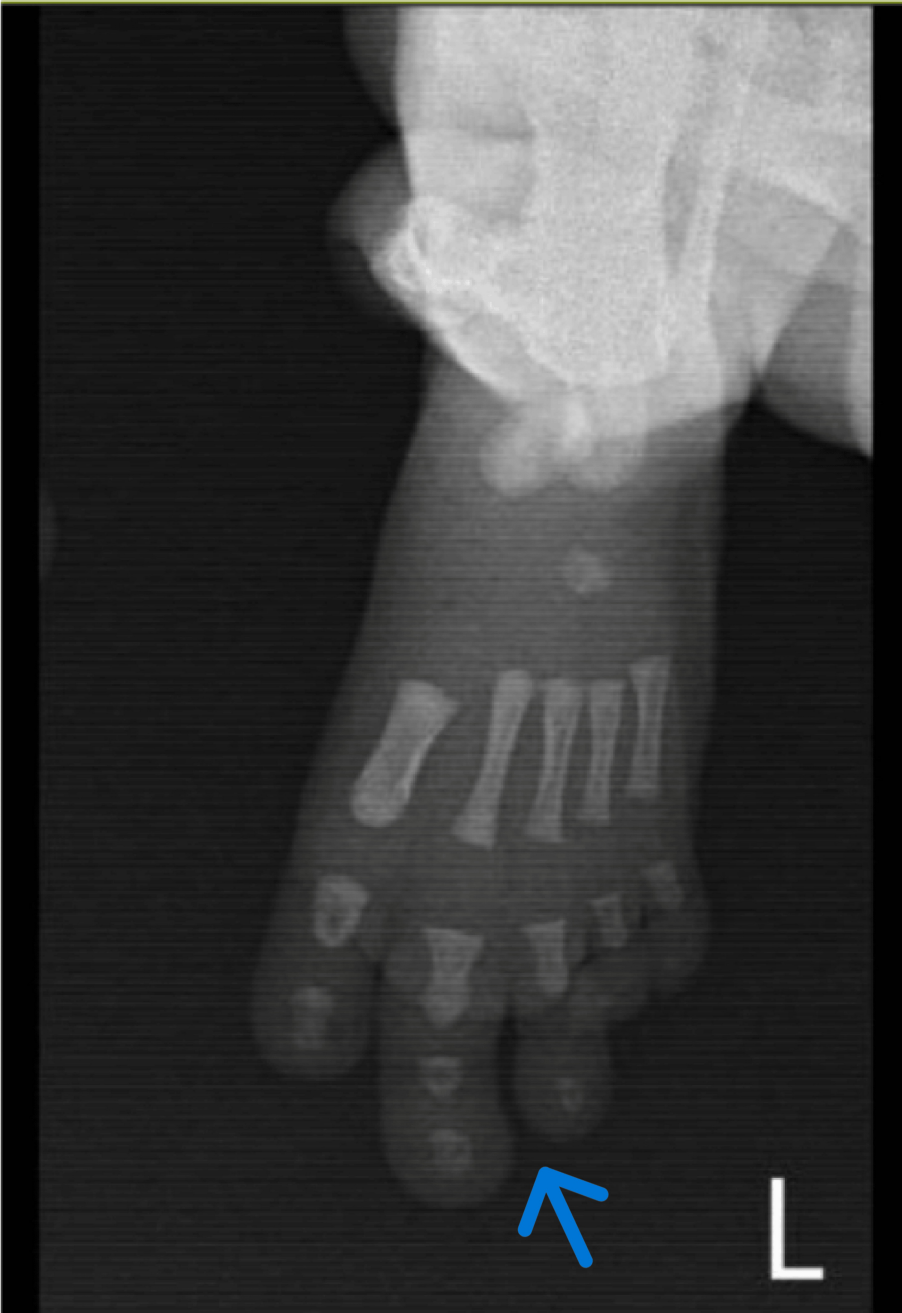
Radiograph of the left foot; enlargement of the soft tissue and bone.

Upon initial physical examination, the newborn exhibited significant asymmetry of the lower extremities, characterized by isolated enlargement of the second and third digits of the left foot (Figures 1-3). No other abnormalities were observed.

Serial measurements of the second and third toes were performed as follows: at birth, 23 mm and 16 mm on the affected (left) foot versus 18 mm and 13 mm on the contralateral (right) foot (Figures 1 and 2); at eight months, 25 mm and 20 mm (contralateral measurements not recorded at this time point); at 18 months, 30 mm and 23 mm; and at four years of age, an overall foot length discrepancy of approximately 6.5 cm (13.5 cm versus 20 cm) was documented.

Imaging demonstrated preserved bone structure and alignment, with no evidence of tumor or soft tissue mass. The length and width of the phalanges of the affected second and third toes were increased in comparison with the contralateral foot (Figure 3).

**Figure 4:**
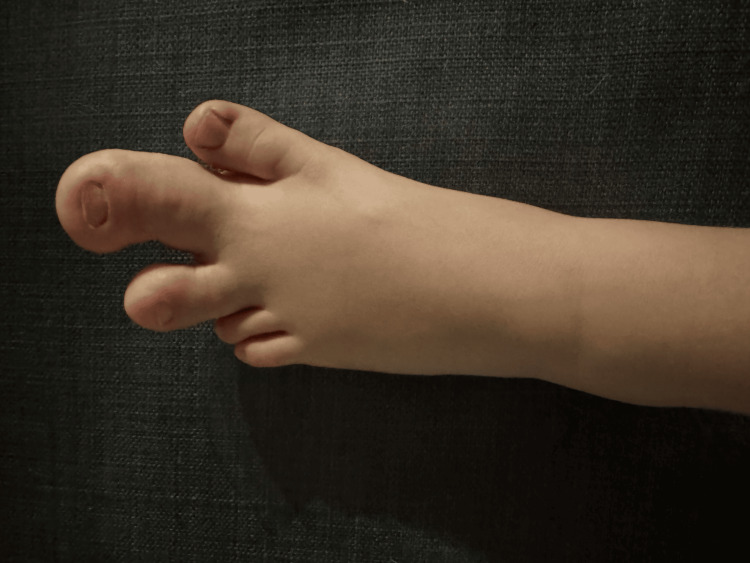
Clinical evolution at 4.5 years old--progressive macrodactyly (superior view); surgical intervention has been proposed and the patient is currently awaiting for Schedule.

**Figure 5:**
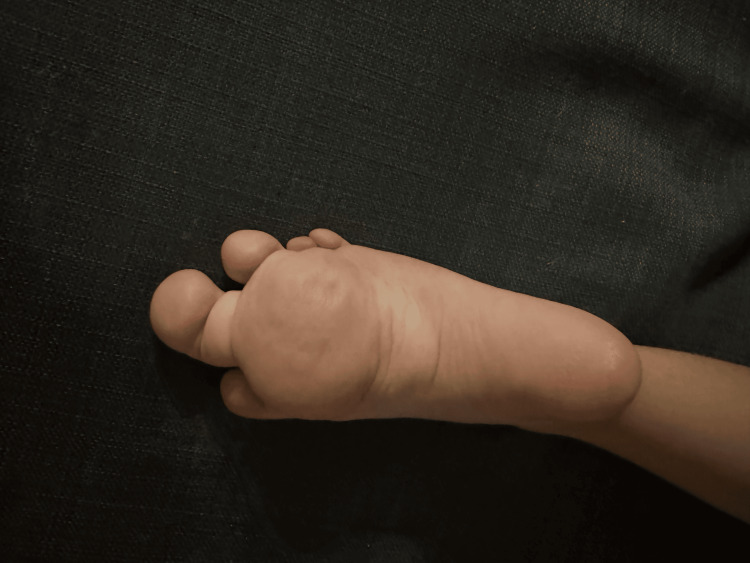
Clinical evolution at 4.5 years old--progressive macrodactyly (inferior view); surgical intervention has been proposed and the patient is currently awaiting for Schedule.

After 4.5 years of follow-up, the asymmetry progressively accentuated (Figures 4 and 5), a clinical course consistent with the progressive type of macrodactyly. The overgrowth had no discernible impact on the child's psychomotor development. The child achieved standing at eight months, supported ambulation at nine months, and independent walking at 14 months. Foot function remained preserved throughout growth, with the exception of a limitation in flexion of the second toe noted at 4.5 years of age, attributed to soft tissue overgrowth on the plantar surface (Figure 5). No pain was reported at any stage. The main clinical concern raised by the family was the need for different shoe sizes for each foot.

The patient was referred for genetic evaluation, and genetic testing was planned; however, the family declined to proceed. No further complementary investigations (ultrasound, magnetic resonance imaging, angiography, or biopsy) were performed.

## Discussion

The clinical presentation in this case is consistent with macrodactyly, specifically the progressive type, given the accentuation of digital asymmetry observed over the follow-up period.

Patient management requires a multidisciplinary approach involving pediatricians, orthopedists, geneticists, physiatrists, and surgeons [2-4]. Diagnostic imaging, starting with plain radiography, is essential to characterize bone involvement [3,4]. Depending on clinical progression, additional modalities--including computed tomography, magnetic resonance imaging, angiography, lymphography, or biopsy--may be indicated to evaluate soft tissue composition and vascularity [3-5]. A primary goal of the diagnostic workup is to establish whether the condition is isolated or part of a syndromic presentation [1-3].

Continuous clinical surveillance is essential to monitor disease progression and assess secondary impacts of overgrowth, including physical discomfort, chronic pain, and functional impairment [2,3]. Regular objective measurements are necessary to evaluate the growth rate [3].

Due to progressive overgrowth, surgical intervention is typically inevitable [2,3,6]. The timing and selection of surgical techniques vary significantly, dictated by the growth rate, functional repercussions, and the surgeon's clinical expertise [2,4,5,7]. The primary goal of treatment is to achieve, through a minimum number of procedures, a pain-free and functional extremity with cosmetically acceptable digits that tolerate standard footwear [5,7,9].

Various surgical techniques may be considered, including soft tissue debulking, epiphysiodesis (to arrest longitudinal growth), osteotomy (for angular correction), amputation, or ray resection [3,4,6,7]. Most children with macrodactyly--particularly those with the progressive type--will require multiple procedures, with the number of interventions ranging from one to six (mean: 2.5) [3,6,7,9]. Functional preservation is a primary concern, alongside aesthetics [6]. Surgical outcomes are generally satisfactory; however, the recurrence rate is high [2].

Maintaining realistic expectations among surgeons, patients, and parents is crucial to preventing disappointment [5,7,9].

## Conclusions

Macrodactyly is a rare but clinically significant congenital malformation that requires early recognition and long-term multidisciplinary management. Pediatricians play a key role as the first point of contact for families, and accurate communication regarding the clinical course, treatment options, and prognosis is essential. In the present case, surgical intervention is indicated primarily for aesthetic purposes, and the patient is currently awaiting the procedure. Consistent with previously published surgical series, outcomes are generally favorable, with acceptable functional and aesthetic results, although multiple procedures are commonly required to achieve satisfactory long-term results.

## Appendices

The authors acknowledge the use of artificial intelligence (AI)-based tools (Gemini 3 Flash, Mountain View, California) to refine technical English. All intellectual content, including study design, data collection, statistical analysis, the interpretation of findings, and final decisions regarding the manuscript content, was conducted and verified entirely by the authors.
